# Cell Biology of Cysteine-Based Molecular Switches

**DOI:** 10.1155/2014/157038

**Published:** 2014-02-06

**Authors:** Christian Appenzeller-Herzog, Kenji Inaba, Agnès Delaunay-Moisan

**Affiliations:** ^1^Division of Molecular & Systems Toxicology, Department of Pharmaceutical Sciences, University of Basel, 4056 Basel, Switzerland; ^2^Institute of Multidisciplinary Research for Advanced Materials, Tohoku University, Sendai 980-8577, Japan; ^3^CEA Life Sciences Division, Oxidative Stresses and Cancer Laboratory, CEA Saclay, 91191 Gif sur Yvette, France

Reversible posttranslational protein modifications form the mechanistic basis for the reception and propagation of biological signals in cells. Besides other modifications such as phosphorylation, acetylation, ADP-ribosylation, and ubiquitylation, reduction-oxidation (redox) processes allow reversible structure-function modulation of proteins, which serve as molecular on-off switches in cell biology. Although many protein-bound amino acids and even the peptide backbone can react with oxidizing metabolites during oxidative stress, only three amino acids adopt reversible redox modifications: cysteine, selenocysteine, and methionine. Among these, cysteine-based molecular switches are by far the most prevalent and best studied. Cysteine switches (or “sulfur switches”) respond in heterogeneous, context-dependent manner to a variety of stimuli (endogenous metabolites, chemicals from the diet, xenobiotics, or air oxidants) by direct modification. Common covalent modifications of cysteines include intra- or intermolecular protein-protein disulfide-bond formation, S-glutathionylation, S-cysteinylation, S-nitrosylation, sulfoxidation, and sulfhydration.

Catalyzed, redox-dependent on-off cycles of cysteine centers in proteins regulate processes as diverse as protein folding, aggregation, and trafficking, enzymatic activity, metal chelation, DNA, RNA, protein, or membrane binding, and channel opening. In this special issue, we have attempted to illustrate the versatility of cysteine-based protein regulation and its impact on the physiology of cells and organisms.

In both the secretory pathway and the mitochondrial intermembrane space (IMS), protein maturation often requires the introduction of disulfide crosslinks to promote or maintain protein structure. During this process known as oxidative protein folding, introduced disulfide bridges can be reshuffled, until the native conformation is achieved. Dedicated oxidative folding catalysts, as reviewed by Y. Onda, exist in the endoplasmic reticulum (ER), IMS, and chloroplasts in plant cells as well as in the extracellular space. The disulfide-generating machineries in ER and IMS are conserved in plants, fungi, and animals. Evolutionary and mechanistic aspects of disulfide-bond formation in IMS are discussed by M. Fischer and J. Riemer. Interestingly, the core components of this machinery, Erv1/ALR and Mia40, have additional, poorly understood functions in liver regeneration and hypoxia response, which are likely fulfilled through mechanisms other than oxidative folding in IMS.

Two contributions are concerned with the involvement of cysteines in the regulation of antibody secretion and differentiation of B lymphocytes. The review article by T. Anelli and E. van Anken enlightens how cysteine redox status acts as a quality control checkpoint to ensure that only mature IgM antibodies leave the compartments of the early secretory pathway en route to the blood stream. Immature antibodies are tagged with a free cysteine, which is covalently trapped by interchain disulfide formation with the retrieval factor ERp44 at the lowered pH of post-ER compartments. In this process, the active site cysteine in ERp44 apparently acts as a pH sensor. Antibody production in activated B lymphocytes is a bulk process, which—besides rigorous quality control—relies on massive ER expansion and disulfide-bond formation. How far the differentiation of resting B cells into antibody-secreting plasma cells affects the overall levels of protein disulfides and of S-glutathionylation relative to the levels of reduced cysteines was addressed by J. R. Winther and I. Braakman and colleagues. They report that ER expansion per se does not lead to a global increase in oxidation of protein-bound cysteines, which is only observed later in differentiation upon induction of antibody synthesis. Furthermore, the fraction of disulfides present as S-glutathione adducts remains constant.

Hydrogen peroxide (H_2_O_2_) is a signaling molecule controlling essential aspects of cellular life and death. Peroxiredoxins are critical H_2_O_2_-reducing enzymes, which shape the amplitude and duration of local accumulation of this second messenger. Importantly, their active site cysteine is inherently prone to H_2_O_2_-driven inactivation by sulfoxidation. As a consequence, H_2_O_2_ signals can transiently override the peroxiredoxin defense and mediate downstream signaling events. The review of S. W. Kang and colleagues focuses on typical 2-cysteine peroxiredoxins and provides a comprehensive overview of their cell biological functions. Interestingly, some of these are also linked to cellular signaling events without being directly related to H_2_O_2_ reduction. On a different note, M. Molin and A. B. Demir address the function of a cytosolic peroxiredoxin in calorie restriction-mediated life span extension. Under these conditions, ATP-driven reactivation of the hyperoxidized form of this peroxiredoxin is stimulated. As the authors hypothesize, this nutrient-sensing mechanism is likely intertwined with other nutrient-responsive processes such as vacuolar proton pumping, mitochondrial function, and iron metabolism. A third contribution to peroxidases by T. Ramming and C. Appenzeller-Herzog summarizes the nonoverlapping functions of the three known ER-resident H_2_O_2_-reducing enzymes, peroxiredoxin IV and glutathione peroxidases 7 and 8.

Finally, two articles—a review by J. D. Atkin and colleagues and a research paper by A. Odermatt and colleagues—represent links of cysteine-dependent regulation with disease. M. Halloran et al. explain how S-nitrosylation and S-glutathionylation of the active site cysteines in protein disulfide isomerase are implicated in various neurodegenerative diseases. L. G. Nashev et al. examine the role of a conserved cysteine in the NADPH-binding region of 17-hydroxysteroid dehydrogenase 1, a prognostic marker for tumor progression and survival of patients with breast cancer and other estrogen-dependent cancers. While mutation of this cysteine does not change the kinetic parameters of the enzyme, its alkylation by sulfhydryl-modifying agents irreversibly inhibits dehydrogenase activity.

